# Citric Acid as a Potential Prostate Cancer Biomarker Determined in Various Biological Samples

**DOI:** 10.3390/metabo12030268

**Published:** 2022-03-21

**Authors:** Magdalena Buszewska-Forajta, Fernanda Monedeiro, Adrian Gołębiowski, Przemysław Adamczyk, Bogusław Buszewski

**Affiliations:** 1 Institute of Veterinary Medicine, Faculty of Biological and Veterinary Sciences, Nicolaus Copernicus University in Toruń, 1 Lwowska St., 87-100 Toruń, Poland; 2 Department of Biopharmaceutics and Pharmacodynamics, Faculty of Pharmacy, Medical University of Gdańsk, 107 Gen. J. Hallera Ave., 80-416 Gdańsk, Poland; 3 Centre for Modern Interdisciplinary Technologies, Nicolaus Copernicus University in Toruń, 4 Wileńska St., 87-100 Toruń, Poland; fernandamonedeiro@hotmail.com (F.M.); adrian.golebiowski@doktorant.umk.pl (A.G.); bbusz@umk.pl (B.B.); 4 Department of Environmental Chemistry and Bioanalytics, Faculty of Chemistry, Nicolaus Copernicus University in Toruń, 7 Gagarina St., 87-100 Toruń, Poland; 5 Department of General and Oncologic Urology, Nicolaus Copernicus Hospital in Torun, 17 Batorego St., 87-100 Toruń, Poland; przemekad@poczta.onet.pl

**Keywords:** cryosections, citric acid, prostate cancer, chemometrics, potential biomarkers

## Abstract

Despite numerous studies, the molecular mechanism of prostate cancer development is still unknown. Recent investigations indicated that citric acid and lipids—with a special emphasis on fatty acids, steroids and hormones (ex. prolactin)—play a significant role in prostate cancer development and progression. However, citric acid is assumed to be a potential biomarker of prostate cancer, due to which, the diagnosis at an early stage of the disease could be possible. For this reason, the main goal of this study is to determine the citric acid concentration in three different matrices. To the best of our knowledge, this is the first time for citric acid to be determined in three different matrices (tissue, urine and blood). Samples were collected from patients diagnosed with prostate cancer and from a selected control group (individuals with benign prostatic hyperplasia). The analyses were performed using the rapid fluorometric test. The obtained results were correlated with both the histopathological data (the Gleason scale as well as the Classification of Malignant Tumors (pTNM) staging scale) and the biochemical data (the values of the following factors: prostate specific antigen, high-density lipoprotein cholesterol, low-density lipoprotein cholesterol, triglyceride, total cholesterol, creatinine and prolactin) using chemometric methods. For tissue samples, the results indicated a decreased level of citric acid in the case of prostate cancer. The analyte average concentrations in serum and urine appeared to be corresponding and superior in the positive cohort. This trend was statistically significant in the case of urinary citric acid. Moreover, a significant negative correlation was demonstrated between the concentration of citric acid and the tumor stage. A negative correlation between the total cholesterol and high-density lipoprotein and prolactin was particularly prominent in cancer cases. Conversely, a negative association between low-density lipoprotein and prolactin levels was observed solely in the control group. On the basis of the results, one may assume the influence of hormones, particularly prolactin, on the development of prostate cancer. The present research allowed us to verify the possibility of using citric acid as a potential biomarker for prostate cancer.

## 1. Introduction

Cancer cells have much more intense growth than normal cells. Consequently, a tumor could be diagnosed by detecting related changes in the metabolic pathways with the use of analytical techniques. Currently, analytical procedures lean towards omics approaches: metabolomics, lipidomics and proteomics. Proteomics is one of the analytical tools widely implemented in tumor diagnoses. In this approach, different sample matrices, such as blood, urine, seminal plasma and exosomes, are found to be suitable, and its application has been deeply discussed. 

However, due to the biodiversity of cancer cells and the use of various analytical techniques, the results obtained within proteomic study are frequently not convergent [1]. For this reason, the application of bioinformatics and statistical analysis is necessary in order to interpret the obtained data. The use of bioinformatics in the case of radiomic, genomic and radiogenomic research for the detection of prostate cancer was reported by Ferro et al. [2].

The results presented by Alkhateeb et al. [3] indicate a correlation between differences in gene expression and cancer stage. The authors indicated that Prostaglandin F receptor (PTGFR), Neuronal regeneration related protein (NREP), Small Cajal body-specific RNA 22 (SCARNA22), Dedicator of cytokinesis 9 (DOCK9), Feline leukemia virus subgroup C cellular receptor family, member 2 (FLVCR2), IKAROS family zinc finger 3 (Aiolos) (IKZF3), Ubiquitin-specific peptidase 13 (USP13) and Cytoplasmic linker associated protein 1 (CLASP1) were found to be potential biomarkers, which may support the diagnosis of cancer. Their determination enables differentiation in accordance with the advancement stage of cancer as well as to evaluate the specificity of the potential biomarkers in order to predict prostate cancer progression [3].

Recently, great interest has been focused on lipidomics. Prostate cancer (PCa) can be considered as a lipid-enriched type of tumor. Fatty particles play a crucial role in cell differentiation and tumor progression. Moreover, increased lipogenesis is observed within carcinogenesis. For this reason, lipids are found to be promising indicators for the detection of PCa in the early stages of the disease [4].

Zhou et al. [5] performed lipidomic analysis on 141 plasma samples. On the basis of the results, followed by the bioinformatic and statistical analysis, 12 lipid species were identified as individual plasma lipid biomarkers of Prostate cancer (PCa) [5]. Buszewska-Forajta et al. used matrix-assisted laser desorption/ionization mass spectrometry (MALDI-MS) technique for the detection of specific indicators of PCa through the use of a lipidomics approach [6]. Owing to the obtained findings, it was observed that prostate cancer appears to be linked both to elevated de novo biosynthesis of steroid hormone-related fatty acids and the lipid metabolism—particularly in mechanisms involving lipid oxidation.

Moreover, phospholipids and fatty acids presented themselves as discriminative features for PCa, indicating potential biomarkers of this type of neoplasm. On the other hand, PCa is known as a hormone-related type of cancer that is regulated by steroid hormones, mainly androgens. Moreover, it is assumed that the development of PCa can be related to prolactin. Prolactin receptors are found in the prostate tissue; increased levels of this hormone in pre-cancerous lesions were reported.

Cerrato et al. (2021) showed significance in detecting zwitterionic and positively charged species in the early diagnosis of prostate cancer [7]. Franz et al. indicated the key contribution of zinc and its transporters in the development and prognosis of PCa. Other substances reported as strongly associated with the development of PCa include citric acid (CA) and its metabolites. 

Monitoring changes in the CA concentration in various biological materials could provide information regarding the molecular mechanisms related to the tumor development. The fast detection of PCa is crucial due to the high mortality associated with this disease among men. The treatment of PCa is found to be challenging, especially in the advanced stages of the disease. In addition, its diagnosis is also problematic due to the asymptomatic development of the disease. For this reason, further knowledge regarding the role of CA in PCa becomes relevant [4,6,8]. 

**Figure 1:**
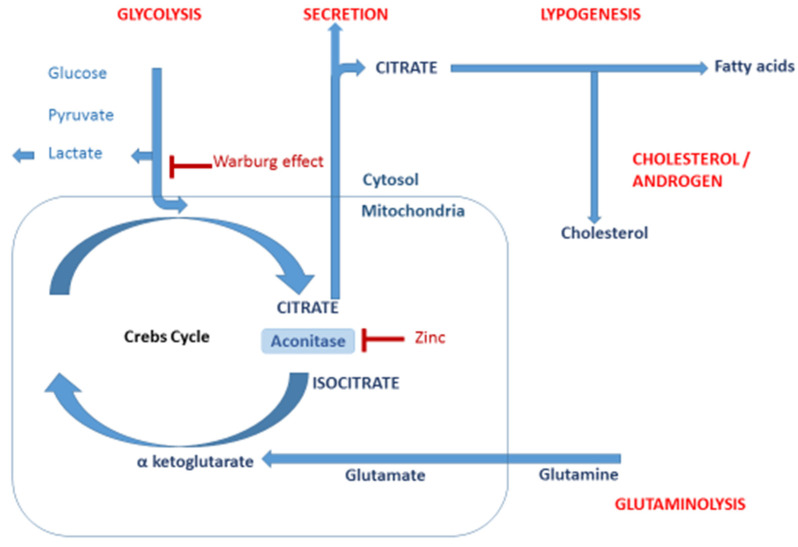
The scheme of the regulated CA pathway (Adapted from [8]).

CA is the main constituent of semen, which is the specific and unique secretion from the prostate gland. Schersten first confirmed the presence of CA in 1929 [9]. Considering all mammals, CA and its structural isomer, isocitrate, have the highest concentration in the prostate when compared with other organs [10]. From a biological point of view, citrate is crucial in the production of metabolic energy and in the synthesis of cholesterol, fatty acids and isoprenoids. These compounds have an essential role in cancer development and progress. Therefore, the determination of CA and other intermediates of the tricarboxylic acid cycle (Krebs cycle) in human tissues is a relevant approach to diagnose a prostate tumor. The scheme of the regulated pathway of the metabolism of CA in a cell is presented in Figure 1.

Spectrophotometric techniques are found to be a promising alternative tool for the quantitative determination of CA. The application of dedicated commercial kits enables the assessment of CA in several biological matrices. Citrate links carbohydrates and the fatty acid metabolism [11,12]. Simultaneous quantitative analyses of intermediates have been conducted using chromatography coupled to mass spectrometry [13,14], nuclear magnetic resonance (NMR) [15,16] and spectrophotometric techniques [17,18]. 

Spectrophotometric techniques can be considered to constitute suitable alternative methods for the quantitative determination of CA. The application of dedicated commercial kits enables the assessment of CA in several biological matrices. The analysis is simple, relatively fast, cheap and reproducible. Considering this, such tests designed for the quantitative determination of CA in various biological matrices appear to be suitable for providing additional information to “*omics*” studies.

The present study aimed to evaluate the role of CA in the pathomechanism of PCa. For this purpose, this compound was determined in three matrices (urine, serum and tissue) with the use of a colorimetric test. Moreover, the results were correlated with other biochemical parameters in order to obtain new insights into PCa molecular mechanisms. To the best of our knowledge, this is the first time for such a type of analysis to be combined with biochemical and histopathological data. The results may explain the changes that occur during the development of the disease, with particular differences between the different stages of the disease.

## 2. Results

### 2.1. Calculation of the Concentration of Citric Acid

The concentration of citrate in samples was calculated in terms of nmol/μL. Considering the molecular weight of CA, the final results are provided in ng/μL of the sample, for urine, serum and tissue extracts. The mean values obtained for all the studied matrices are listed in Table 1. The average concentrations of the evaluated parameters, displayed according to the study subgroups, are shown in Table 2.

### 2.2. Statistical Analysis

**Figure 2:**
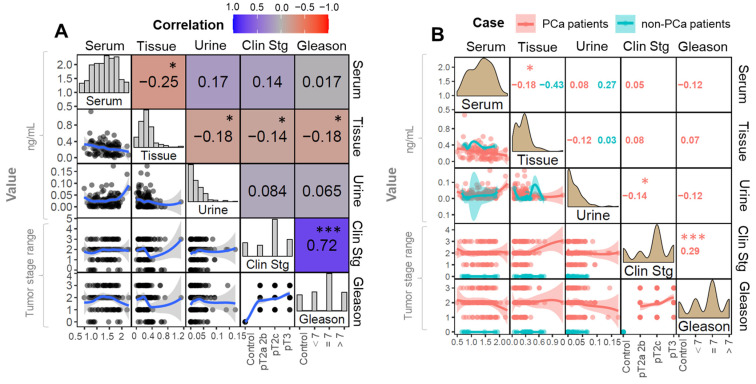
Plots showing the correlations between citric acid levels measured in the different biological matrices and their relationship with cancer staging (in terms of clinical stage and GS)—with the respective correlation coefficients calculated on the basis of (**A**) all cases or (**B**) separately, according to the study groups. Numerals inside the boxes refer to the Spearman coefficient (rho) found for the given bicorrelation. The symbols “***” and “*”, correspond to *p* < 0.001 and ≤0.05, respectively. Clin Stg = cancer clinical staging.

**Figure 3:**
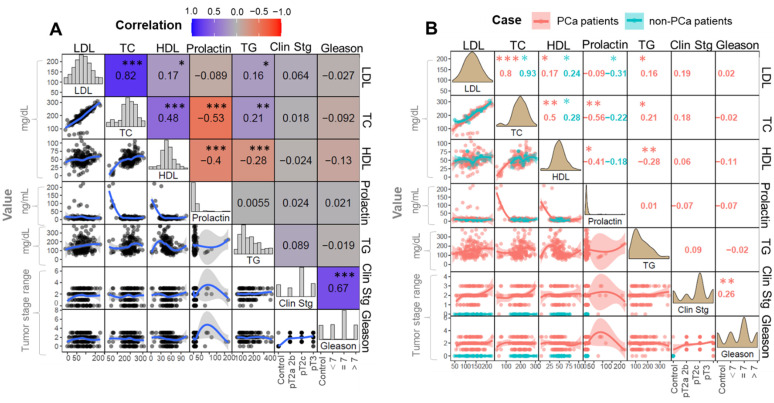
Plots showing the correlations between biochemical parameters in blood and cancer staging (in terms of clinical stage and GS)—with the respective correlation coefficients calculated based on (**A**) all investigated samples or (**B**) separately, according to the study groups. The numerals inside the boxes refer to the Spearman coefficient (rho) found for the given bicorrelation. The symbols “***”, “**” and “*”, correspond to *p* < 0.001, ≤0.001 and ≤0.05, respectively. LDL = low density lipoprotein, TC = total cholesterol, HDL = high density lipoprotein, TG = triglycerides and Clin Stg = cancer clinical staging.

The correlation matrix presents the dependence between multiple parameters simultaneously. The rows and columns of the matrix detail the investigated variables, and each corresponding cell shows the result of a correlation test performed between these features. Multiparametric correlation plots displaying the relation between variables are presented in Figure 2 and Figure 3. In these, the distribution of variables is shown on the diagonal of the matrix. On the left diagonal, there are bivariate plots with a fitted line. The numerals in the right diagonal and the cell color code represent the correlation coefficient and the encoded statistical significance, respectively. 

The performed correlation analysis demonstrated that the concentration of CA in tissue was negatively correlated to the progression of cancer clinical staging (rho = −0.14) and Gleason scale (GS) (rho = −0.18). Although weak, these correlations were statistically significant (*p* ≤ 0.05) (Figure 2A). This demonstrates that CA levels tend to be lower in PCa tissue samples; However, since the displayed coefficient is much lower than [0.9], a decrease in CA cannot be directly associated with a progression in the PCa stage. For this reason, according to the present results, it is unlikely that CA concentrations in tissue alone may be used to derive the corresponding PCa stage. 

To summarize, these statistically relevant correlations appear to be mostly connected with a significatively lower concentration of CA in positive PCa tissue in relation to control samples. Additionally, a weak negative correlation between CA levels in tissue and serum was found (rho = −0.25). A correlation of the exact nature between CA in tissue and urine was reported (rho = −0.18). Solely in positive cases, a negative (although weak and significant) association between CA in tissue and serum was detected (Figure 2B).

The multiparametric correlation analysis was also performed on blood analysis data (biochemical parameters). Prolactin levels appeared to be negatively correlated with the total cholesterol (TC, rho = −0.53) and high-density lipoprotein (HDL, rho = −0.40), at moderate extension (Figure 3A). The second approach (Figure 3B) revealed that some correlations were characteristic for the control and cancer group. The observed negative moderate correlation between TC or HDL and prolactin was much more significant in cancer cases (rho = −0.56 and −0.41 in the positive cohort, against rho = −0.22 and −0.18 in the control group).

Additionally, a negative correlation between low density lipoprotein (LDL, rho = −0.31) and prolactin concentration was revealed, with a relevant strength exclusively in the control cohort. However, in both approaches, the biochemical parameters were not found to be related to the stage of PCa.

PCA biplots were built in order to evaluate data patterns considering the multiple parameters that were analyzed. The orientation and lengths of arrows represent the extension of the influence of a certain variable (vectors) on principal components. Vectors plotted closely (between each other or adjacent to certain features) represent more correlated parameters, while those presenting opposite directions can be considered as negatively associated. Therefore, it is possible to observe that CA levels in tissue appear negatively correlated to the trend observed for this variable in urine and serum (these last two presenting considerable correspondence between each other). 

**Figure 4:**
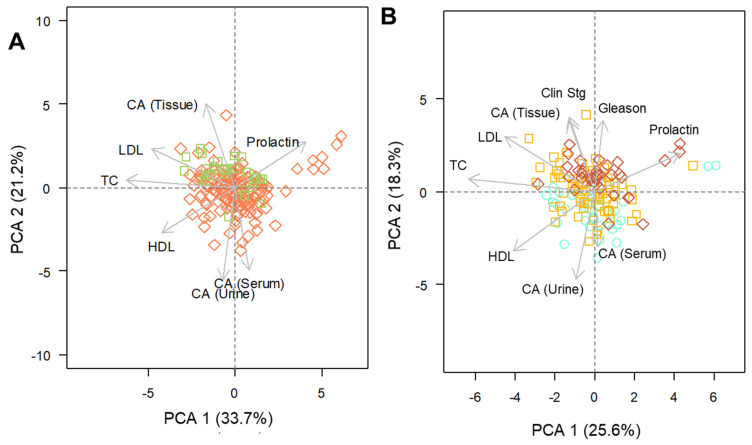
PCA correlation biplot of data comprising the evaluated parameters for (**A**) both groups and (**B**) within positive class. In (**A**): green squares = control cases and red diamonds = positive cases; In (**B**): green circles = GS < 7, yellow squares = GS equal to 7 and red diamonds = GS > 7. CA = citric acid, LDL = low density lipoprotein, TC = total cholesterol, HDL = high density lipoprotein and Clin Stg = cancer clinical staging.

Moreover, a positive association between CA measured in tissue extracts and control cases (a respective arrow plotted in the vicinities of dots representing non-cancer cases) is indicated. A negative association between the variance in the levels of prolactin and HDL, TC and LDL is also depicted (Figure 4A). Analyzing solely positive cases, a relationship between the variables associated to cancer staging and analyte levels in tissue (Figure 4B) may be detected. Additionally, greater values for CA in serum and urine (as well as HDL levels) appear particularly connected with earlier cancer stages (Gleason < 7). Conversely, later stages (Gleason > 7) appear to be linked to greater levels of the analyte in tissue along with higher LDL and prolactin levels.

**Figure 5:**
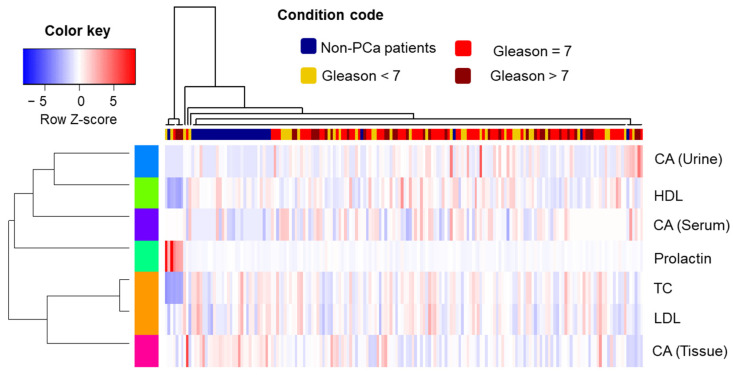
HCA associated with a heatmap; The condition code refers to different PCa stages according to GS. CA = citric acid, LDL = low density lipoprotein, TC = total cholesterol and HDL = high density lipoprotein.

The results of Hierarchical Clustering Analysis (HCA) conducted on data organized according to GS values are presented in Figure 5. The dendrograms represent the level of similarity between cases or features. The control samples remained concentrated in an isolated cluster, indicating the differential response of evaluated parameters for PCa and non-PCa samples. On the other hand, positive samples could not be differentiated based on the parameter distribution (PCa cases belonging to equivalent GS ranges were not grouped together). 

Regarding the studied variables, a congruent behavior between TC and LDL values was observed, once these features were assigned to the same cluster. TC, LDL and CA measured in tissue were the variables where trends across the samples appeared to be more similar, while the remaining parameters composed a different hierarchical group. The results presented by HCA can be considered as concordant with those found in the PCA approach.

**Figure 6:**
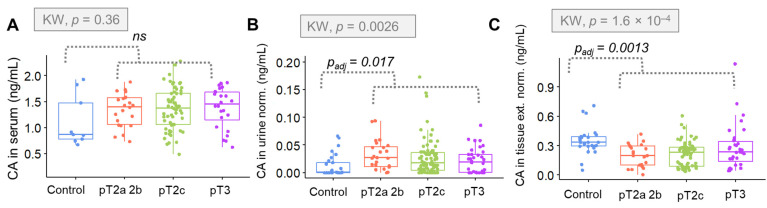
Box plots showing the comparison between the means of citric acid levels determined in (**A**) serum samples; (**B**) urine samples and (**C**) tissue extracts, according to the cancer clinical staging. CA = citric acid, KW = Kruskal–Wallis test, *p*_adj_ = adjusted *p*-value according to the B-H method for the referred comparison, as reported by the Mann–Whitney test, ns = non-significant, norm. = normalized to creatinine (in the case of urine) or to weight (in the case of tissue) and ext. = extract.

**Figure 7:**
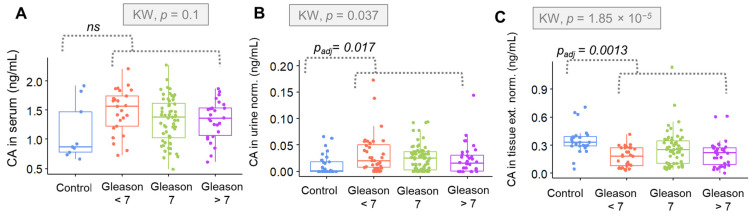
Box plots showing the comparison between the means of citric acid levels determined in (**A**) serum samples; (**B**) urine samples and (**C**) tissue extracts, according to the GS. CA = citric acid, KW = Kruskal–Wallis test, *p*_adj_ = adjusted *p*-value according to B-H method for the referred comparison, as reported by the Mann–Whitney test, ns = non-significant, norm. = normalized to creatinine (in the case of urine) or to weight (in the case of tissue) and ext. = extract.

The distribution of CA levels is depicted using box plots. The variable distribution is shown there according to data groups, comprising controls and different stages of PCa—presented as the clinical staging (Figure 6) and GS (Figure 7). Statistical differences between investigated groups were pointed out by the Kruskal–Wallis test only in the case of CA in urine and tissue samples. Differences in urinary CA were especially significant when analyzing data partitioning according to the clinical staging (*p* = 0.0026), rather than the GS (*p* = 0.037). 

The urinary concentration of CA was significantly greater for overall cancer groups (*p*_adjusted_ = 0.017). In tissue samples, CA levels were significantly lower in the positive cases (*p*_adjusted_ = 0.0013). The overall distinction between groups was particularly sensitive for the data arranged in accordance with the GS (*p* = 1.85 × 10^−5^, while for the clinical staging, *p* = 1.6 × 10^−4^). When the positive class was divided regarding the varied ranges of GSs, significant differences were found between the intermediary score and the remaining classes (*p*_adjusted_ = 0.038), showing that the CA concentration was relevantly higher when the GS was equal to 7.

## 3. Discussion

The characteristic features of the ideal disease indicators include specificity for the PCa, accuracy to diagnose PCa at any stage of the disease development, along with distinguishing PCa from indolent cases. Moreover, ideal biomarkers should be easy to determine in minimally invasive matrices, such as blood and urine [19]. Recent advances in metabolomics, genomics and proteomics have provided knowledge regarding new potential biomarkers of PCa. One of the recent approaches is the application of combining the multiple gene analysis, such as SelectMDx or Mi Prostatescore for PCa prediction [19,20]. 

Another tool that can be implemented to increase the specificity of PCa diagnoses is the analysis of exosomes (Exos), which may play an important role in cancer pathophysiology [19]. The determination of molecular factors, such as peptides, low molecular weight compounds or lipids with the use of analytical techniques may also be applied. The United States FDA approved three promising biomarkers of PCa, including the urinary Progensa Prostate Cancer Antigen 3 (PCA3) assay as well as a serum prostate health index (PHI) assay and the four-kallikrein (4Kscore).

Moreover, small molecular weight compounds, known as metabolites, should be also highlighted. One of the metabolites that can be regarded as a specific indicator of PCa is CA. Studies in recent years have shown the participation of the CA cycle in the development, proliferation and differentiation of malignant cells of prostate tissue [21,22,23]. It can be assumed that CA may be a potential specific indicator of PCa. 

In this study, the concentration of this metabolite was determined in three biological matrices. First of all, in tissue, as the immediate focus of the disease. For this reason, it can be assumed that the changes are the most significant and reliable in the case of tissue. However, this metabolite was also investigated in the blood and urine in matrices to study the general changes in the body. The noninvasive, sample collection and the relatively unlimited access to matrices can be mentioned as an advantage. Moreover, the present research allowed verification of the usefulness of CA as an early indicator of PCa.

The results of our study demonstrated that, in the case of tissue samples, the concentration of CA significantly decreased with the progression of malignancy: we observed that the concentration of CA in tissue was negatively correlated to the pTNM staging and GS in a statistically significant manner. Our results are in accordance with the studies conducted by [21,23]. Carcinogenesis can be characterized by the increased proliferation of cancer cells. 

On the one hand, growing cancer cells require high amounts of carbon species to produce all structural and nutritious compounds. Cell proliferation and their differentiation during cancer development causes morphological changes in the structure of the cell. As a consequence, the structure of the cell membrane is loosened, which prevents the storage of zinc ions inside the cell [21,22]. This leads to the activation of aconitase, which is active in the prostate cell. 

Such a process is triggered by the waste of zinc ions (an essential micronutrient), which inhibits mitochondrial (m-) aconitase. The aconitase enzyme catalyzes the first step of the Krebs cycle, consisting in the oxidation of citrate to isocitrate [20]. In this case, a major shift in energy metabolism occurs, and an increased activity of CA cycle is found in comparison to benign cells. Finally, the CA is transformed to isocitrate, which leads to a significant reduction of CA in the cell [8,22].

Going further, alterations in the concentration of CA in relation to PCa stage were evaluated. The results indicated that CA levels in tissue are particularly sensitive to the clinical staging and specific classification made in relation to the prostate (GS). In the case of clinical staging criteria, a positive correlation was observed. The higher the degree in the pTNM scale, the lower the concentration of CA. 

In the case of the Gleason evaluation, the concentration of CA was up to Gleason = 7, and then a plateau or even a decrease was observed. These results reflect the mechanism proposed by Costello et al. [22]. The pTNM scale refers to the anatomic extent of the disease. It describes the degree of invasion in terms of the tumor, lymph nodes and margins. This is a general assumption that does not reflect changes at the morphological level of the cell, with a particular emphasis on differentiation. A more detailed description is possible with the Gleason scale evaluation, being more related to the molecular characterization of the tumor with a special emphasis on cell differentiation. 

The results indicate that there is a strong proliferation of cells with a simultaneous inhibition of aconitase in the initial stage of the development. With the differentiation, the membrane disintegrates, which results in the loss of the ability to accumulate zinc ions and leads to the activation of aconitase. As a result, CA is a subtraction of the transformation to isocitrate. Finally, the concentration of citrate decreases with a simultaneous increase in isocitrate in the cell. 

The intensity of changes is closely correlated with the degree of differentiation of neoplastic cells. The Gleason grading system can easily evaluate the differentiated morphology. Ren et al. showed that citrate can inhibit tumor growth in diverse tumor types and via multiple mechanisms by citrate supplementation [24]. Caiazza et al. studied the long-term effects of citrate supplementation on PCa cells; they demonstrated that a chronic citrate administration might select for resistant cells [25].

Additionally, the obtained results indicate that tissue can be considered as the most reliable matrix to assess CA to derive a PCa diagnosis. The use of tissue allows for the direct assessment of the tumor environment and cancer cells. In our study, tissue was also found as the most discriminative matrix. Therefore, the use of tissue for the determination of CA for PCa diagnostics has clinical application potential.

In the case of urine and serum samples, the opposite correlation was found. In both matrices, the concentration of CA in the control samples was lower than in the samples from patients with PCa. In the above mentioned matrices, the concentration of citrate is associated with changes occurring throughout the body rather than directly at the focus of the disease. 

Nevertheless, the concentration of CA in urine samples was significantly greater for the overall cancer group (*p* = 0.011). Moreover, a correlation with the stage of cancer was shown, especially regarding the clinical staging (*p* = 0.0014). Considering the GS (*p* = 0.02) the correlation between this parameter and the concentration or urinary CA was significantly weaker. Statistical significance found in urinary CA suggests that this is a promising approach for non-invasive and fast screening for PCa detection. These biochemical changes may be useful in the context of a rapid diagnosis of PCa.

In the case of serum samples, the concentration of CA was higher for the cancer group in general; however, these changes were not assigned as significant. Moreover, the correlation between the citrate concentration and clinical stage/GS was evaluated. We observed that the levels of CA were higher for PCa samples in comparison to control group. The concentration of CA was higher for the PCa group, but no significant changes in the concentration of citrate were observed according to the clinical stage of the tumor. 

The relation was slightly different when the concentration of serum CA and the Gleason scale were considered: the concentration of citrate was higher in the case of PCa samples; nevertheless, we observed that, as the tumors progressed, the concentration of citrate decreased. This observation is complementary to the tissue data and can be easily explained by the similar complexity of these two matrices, i.e., tissue and blood. Within the study, the correlation between CA and other molecular indicators was analyzed. Due to the fact that the prostate is a hormone-related gland, the influence of hormones on PCa pathogenesis may be significant. 

Normal prostate metabolism is coordinated by androgen receptor signaling and characterized by a physiological truncation of the Krebs cycle to enable the production and secretion of citrate into prostatic fluid [26,27,28]. It was reported that androgens, which bind to androgen receptors, influence prostate cancer development. Although the pathomechanism remains unknown, recent studies indicate the relationship between the level of androgens and cholesterol [27,29]. 

Undoubtedly androgens stimulate the lipogenic enzymes that cause the increased synthesis of fatty acids and cholesterol. For this reason, searching for the correlation between the cholesterol and other molecular factors may provide significant knowledge in terms of PCa development and progression.The role of cholesterol in this mechanism can be considered in two ways. First of all, cholesterol is the initial metabolite of hormone-related metabolic transformation pathways. On the other hand, cholesterol can be considered a nutrient that enters the body with food. Its concentration can be related with the patients’ lifestyle in terms of diet. 

Therefore, recent references suggest that diet and especially consumed fats may play a role in the development and progression of prostate cancer. Cholesterol can be determined with regular tests provided by the clinical laboratories. By monitoring changes in cholesterol and its fraction levels, the hypothesis on its influence on cancerogenesis may be verified. Our results provide knowledge about the increased level of cholesterol and HDL fraction in the case of PCa patients. The opposite phenomena were observed in the case of the control group, where the LDL fraction increased. Heir et al. [30] reported that elevated level of lipids, including free cholesterol, were associated with PCa development [30]. 

In contrast, the meta-analysis reported no significant correlation between the total cholesterol fraction and the corresponding PCa staging [31]. These results are in accordance with the results presented by us. When considering the part played by cholesterol in the PCa development and progression, the role of prolactin should also be taken into account. Prolactin is an anterior pituitary hormone that influences the function of several organs; therefore, this hormone may be significant in the regulation of prostatic cholesterol, especially free cholesterol, by the stimulatory effect of androgens [32,33].

According to the literature, prolactin stimulates prostate growth activity, possibly due to its association with the prostate cell proliferation and the regulation of the angiogenesis process [34]. It was found that prolactin receptors are present in prostate tissue. Moreover, the precancerous state of prostate tissue contains an increased amount of these types of receptors. For this reason, prolactin is believed to be involved in the neoplastic process. The obtained results show normal prolactin levels in both groups; however, in the case of PCa patients, the level of prolactin was higher (9.53 ng/mL) than in the controls (8.51 ng/mL).

Additionally, it was observed that the level of prolactin was not associated with the stage of cancer. Similar results were obtained by [34]. The authors reported that circulating prolactin was not correlated with an increase in the PCa risk. The influence of prolactin on the PCa development was the aim of the study conducted by [35]. The study was performed with murine model transgenic mice. 

The results provide the knowledge that the overexpression of prolactin induces the disorganized amplification of the basal/stem cell compartment, which is found to be tumor-initiating cells in the prostate. The influence of prolactin on PCa development is still unknown. For this reason, there is a need to conduct research on a large set of samples, with special emphasis placed on the role of androgens. As the androgen receptor regulates the prostate, its role may be greater than expected in the neoplastic process.

## 4. Materials and Methods

### 4.1. Biological Material

The participants enrolled in the study were recruited by a specialist of urology from the Nicolaus Copernicus Specialist Municipal Hospital (Toruń, Poland). Patients were matched in terms of age and body mass index (BMI). A total of 184 biological samples were collected from individuals with PCa (*n* = 154) and control subjects (*n* = 30). All diagnoses, both PCa and benign prostatic hyperplasia (BPH), were histologically confirmed by prostate biopsy. Blood biochemical indicators of all participants were obtained within the physical examination. 

Basic laboratory tests were performed to determine the general condition of the patient qualified for the study. Relevant information, such as age and weight as well as levels of the prostate specific antigen (PSA), high-density lipoprotein cholesterol (HDL), low-density lipoprotein cholesterol (LDL), triglyceride (TGs), total cholesterol (TC), creatinine (CREA) and prolactin (PRL) were obtained. Patients from the PCa group presented elevated PSA levels during screening tests and positive assessment in ultrasound and digital rectal examinations. 

Each patient had their PSA level determined according to the routine laboratory protocol. The mean PSA concentration in the PCa group was 14.80 ± 12 ng/mL (max. 45.53 ng/mL and min. 0.008 ng/mL). The control group consisted of patients with diagnosed BPH. In this group, the average concentration of PSA was relatively lower (6.34 ± 3.82 ng/mL, max. 18 ng/mL and min. 0.868 ng/mL). Non-cancer patients enrolled in the control group were declared to be in good health condition. 

All participants gave signed written informed consent forms and filled a standard questionnaire. The study was conducted in accordance with the Declaration of Helsinki Ethical Principles. The Independent Commission positively reviewed the research for Bioethics Research, Medical University of Gdansk (number of consent: NKBBN/432/2016). All experiments were performed by following the relevant guidelines and regulations. Three types of biological materials (tissue, blood and urine) were collected from each patient.

In the case of PCa patients, the tissue was collected during radical prostatectomy surgery, while control tissue was obtained during biopsy. In the case of the control group, the presence of cancer cells was ruled out owing to the histopathological evaluation. The tissue was preserved in accordance with recommended diagnostic histopathology protocols, based on fixation and paraffin embedding procedures (https://www.leicabiosystems.com/knowledge-pathway/an-introduction-to-specimen-preparation/, accessed on 17 February 2022). A detailed description is presented in Buszewska-Forajta et al. [4]. 

The diagnosis was confirmed by the histopathological evaluation, where tissue samples were characterized in reference to the Gleason scaling. Additionally, the cancer staging was evaluated in accordance with the pTNM scale. Patients were divided into subgroups according to the histopathological assessment. The first subgroup corresponds to samples where cancer was detected only in one side of the prostate (pT2*). The second subgroup, including the most common stage pT = 2, is characterized by the presence of a tumor involving both lobes (pT2**). Other cases were described as pT3, including those found as tumors extending through the prostatic capsule.

The summarized information about the collected samples, divided in reference to each parameter, is listed in Table 3 and Table 4. Table 3 shows subgroups divided in accordance with the Gleason scale, while Table 4 presents subgroups classified in relation to the description of the primary tumor.

The formalin-fixed, paraffin-embedded (FFPE) blocks containing tissue samples were stored at room temperature (20–22 °C). Prior to the analysis, FFPE blocks were cut to obtain 20 µm sections, which were implemented to the qualitative analysis of CA. Regarding the remaining matrices, 1.5 mL of morning urine and serum samples were collected in Eppendorf tubes and kept frozen at −80 °C until the analyses were conducted. The other two biological materials, namely urine and blood, were collected before the biopsy.

Blood samples were collected from each of the participants enrolled in the study. Due to the daily changes in hormone levels, each sample was drawn from fasting patients, at the same time period of the day (7–9 AM). Venous blood was collected into two polypropylene tubes containing Ethylenediaminetetraacetic acid (EDTA). Within 2 h to 4 h, blood samples collected “on clot” were centrifuged at the speed of 2500 rpm for 15 min for the collection of serum. Sera were transferred to Eppendorf tubes and stored at −80 °C up to the day of citric acid determination. The second tube of venous blood was used to determine parameters, such as the total cholesterol (TC), LDL, HDL, prolactin (PRL) and triglycerides (TG). These measurements were performed in the hospital laboratory, following routine standard protocols.

Additionally, morning urine samples were collected and vortex-mixed during 15 s. The pH of each urine sample was measured. the urine pH in the control group ranged from 5.0–5.5. In the prostate cancer group, pH values were relatively higher (5.5 to 6.2).

Next, creatinine levels were determined with the use of the routine protocol applied in the hospital laboratory. All tests were performed in accordance with the relevant guidelines and regulations. Collected urine samples were divided into probes and stored at −80 °C until analysis. On the day of the analysis, samples (urine and serum) were thawed on ice for 2 h, centrifuged and filtered.

### 4.2. The Chemicals

The citrate assay kit was obtained from Sigma Aldrich (Steinheim, Germany). Deparaffinization solution was purchased from Qiagen (Bedford, MA, USA).

### 4.3. Sample Preparation

#### 4.3.1. Citrate Reaction Mix

The CA determination in samples was performed with the use of the commercially available Citrate Assay kit. A citrate reaction mix was prepared following the manufacturer’s protocol. Briefly, 44 μL of citrate assay buffer was added to 2 μL of each of three buffers: citrate enzyme mix, citrate developer and citrate probe. Then, 50 μL of such a solution of the citrate reaction mix was added to 50 μL of each regular or standard sample. In the case of blank samples, the prepared composition was slightly different: the citrate reaction mix was prepared by adding 46 μL of the citrate assay buffer to 2 μL of both the citrate developer and the citrate probe.

#### 4.3.2. Standards

Standard solutions were prepared as described by the protocol included in the assay kit. Briefly, 0, 2, 4, 6, 8 and 10 μL of the 1 mM citrate standard were transferred to a 96-well plate. In the next step, the citrate assay buffer was added to each well in order to reach a final volume of 50 μL. The samples were incubated for 30 min at room temperature in the dark. A sample consisting solely of 50 μL of buffer was used as a blank.

#### 4.3.3. Biological Samples

##### Tissue Samples

The deparaffinization procedure was conducted as indicated by the manufacturer of the deparaffinization solution (Qiagen, Germantown, MD, USA). Briefly, the section was placed into an Eppendorf tube, and then 320 μL of deparaffinization solution was added. Next, the sample was vortexed vigorously for 10 s and incubated for 3 min, at 56 °C. Each sample was cooled to room temperature and centrifuged for 1 min at 11,000× *g*. The paraffin-containing solution was decanted, and the clean tissue was transferred to an Eppendorf tube. 

Each section was weighted. The tissue section was homogenized with 100 μL of citrate assay buffer using a Bullet Blender Storm homogenizer (Next Advance, New York, NY, USA). Homogenization was achieved within three cycles. Each homogenization cycle was performed for 1 min, followed by cooling in ice for another 1 min. In the last step, the homogenized sample was centrifuged for 15 min at 15,000× *g*, and 50 μL of the supernatant was transferred to a 96-well plate. Then, 50 μL of the reaction mix solution was added to each well and mixed using a horizontal shaker. The plates were incubated (30 min, in the dark and at room temperature) and then immediately assayed.

##### Urine Samples

Urine samples were prepared following the tissue preparation procedure. Briefly, 50 μL of urine was added to 50 μL of the reaction mix. The sample was vortexed and incubated for 30 min.

##### Serum Samples

Fifty microliters of serum were added to 50 μL of the reaction mix. Similarly, the sample was vortexed and incubated for 30 min.

### 4.4. Instrumentation and Metabolic Profiling

The quantitative analysis of CA in the biological matrix was conducted using the Varioskan™ LUX multimode microplate reader (Thermo Scientific, Waltham, MA, USA). The concentration of CA was calculated on the basis of the intensity of the generated fluorometric product, generated after the interaction with the enzyme. The response measurement was performed at the following wavelengths: λ_excitation_ = 535 nm and λ_emission_ = 587 nm. Each sample was analyzed in triplicate. The values obtained for both regular and standard samples were corrected by subtracting the readings obtained for the blank samples (only buffer with no addition of the citrate standard).

The concentration of citrate was calculated according to the following equation:

S_a_/S_v_ = C, T = *const* (1)

where S_a_ is the amount of citrate in the unknown sample (in nmols) given by the standard curve, while S_v_ corresponds to sample volume (μL) added into the wells.

### 4.5. Statistical Analysis and Validation

The obtained datasets were normalized accordingly. In the case of tissue, the data were normalized in accordance with the weight of tissue. In the case of urine samples, the data were normalized by the creatinine value determined in each analyzed sample. The normality of the data distribution was assessed using Kolmogorov–Smirnov and Shapiro–Wilk tests, employing IBM SPSS Statistics; version 24 (BM Corp., Armonk, NY, USA). The following methods were performed in R environment, using RStudio 1.1.463 console (Boston, MA, USA).

The correlation analysis was evaluated using Spearman’s rank correlation coefficient (rho) in order to assess associations existing between the levels of CA found in the different matrices, biochemical parameters and cancer staging. Plots were generated using “ggplot2” package, with the “corrmorant” extension applied to create customized correlation matrices. The Spearman method allows evaluation of the nature of the connections existing between two sets of data. It assumes a monotonic relationship between two parameters of interest. 

The strength and the direction of a correlation is provided by the calculated Spearman’s coefficient. This is a non-parametric approach and therefore indicated in the case of data with non-normal distribution and/or measured in different scales. The principal components analysis (PCA) was performed using the “prcomp” function. For this method, only data corresponding to at least 10% of the total abundance was included, and missing values were imputed by the variable’s means.

In addition, the data were scaled prior to the analysis. PCA is a multivariate approach that works in reducing the dimensionality of attributes from datasets containing numerous variables. This statistical method permits the visualization of data from a more informative perspective, allowing the observation of relationships existing between all parameters in a comprehensive manner, thus, facilitating the recognition of latent patterns within the data.

Hierarchical cluster analysis (HCA) combined with a heatmap was produced using the “gplots” package, applying z-score normalized data as input. Spearman correlation coefficient was used to calculate the cluster distance, and the unweighted pair group method with the arithmetic mean (“average” method) was used as the clustering criterion. The Kruskal–Wallis test was used to indicate differences among study groups (controls and different stages of cancer) according to the levels of CA found in the biospecimens. 

**Figure 8:**
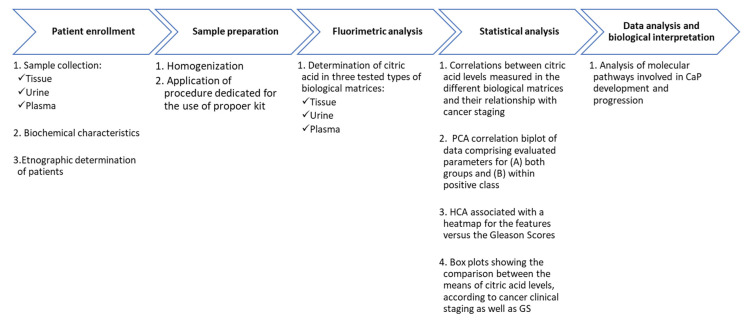
Schematic view of the workflow with special emphasis on the used method.

The Mann–Whitney test was applied to investigate further differences between the control cohort and the positive sub-groups. Box plots were generated with the aid of the “ggpubr” package. The adjustment of the *p*-value was performed using the Benjamini–Hochberg (B-H) procedure. For the statistical analysis, cancer staging parameters were properly encoded as ordinal variables, as follows: control cases = 0, pT2a 2b = 1, pT2c = 2 and pT3a, b or c = 3; Gleason scores (GSs) lower than 7 = 1, scores equal to 7 = 2 and scores greater than 7 = 3. The general workflow of the applied methods is presented in Figure 8.

## 5. Conclusions

In this study, fluorometric analysis was conducted as a rapid and sensitive method to analyze the CA in three biospecimens collected from patients with PCa and non-cancer controls. The application of the commercial kit was demonstrated to be an approach that was selective for the citrate acid determination and universal in terms of the applied matrices. The application of biostatistics provided information about the role of CA in PCa development. 

In summary, the method proposed in this study enables an effective, simple and fast identification of CA in several biological materials. The proposed approach appears to be a promising tool in the diagnostics and prognosis of PCa through the determination of CA. Unfortunately, it appears that the obtained results prove the low specificity of this potential biomarker for matrices, such as blood and urine. Therefore, the determination of this metabolite in the above-mentioned matrices may be insufficiently specific. Considering the mechanisms described above, the determination of both citric acid and isocitrate should be considered. 

The transformation of these metabolites is closely correlated with the development of prostate cancer. In this respect, the developed rapid method for the determination of these potential biomarkers is undeniably a promising and desirable analytical tool. In addition, the determination of the citric acid and/or the citric acid isocitrate panel in semen is noteworthy. Our research has shown that semen is one of the promising matrices, and its composition is, in a way, a metabolic reflection of the processes taking place in the gland. For this reason, the determination of citric acid in semen may have a key impact on improving the diagnosis of prostate cancer in the early stages of the disease.

## Figures and Tables

**Table 1:** The average concentration of citric acid arranged regarding three studied matrices. The table shows non-adjusted *p*-values referring to a comparison between the concentration of parameters in the control and positive groups. * = concentration in the extract, normalized by weight; ** = concentration normalized to creatinine; SD = standard deviation.

Matrix	Mean Concentration of Citrate ± SD [ng/ml]	
	Control Group	PCa Patients
Urine **	0.012 ± 0.002	0.03 ± 0.03
Serum	1.10 ± 0.48	1.40 ± 0.37
Tissue *	0.35 ± 0.14	0.23 ± 0.16

**Table 2:** The average concentration of citric acid and biochemical blood parameters in samples, arranged regarding evaluated subgroups. The table shows non-adjusted *p*-values referring to a comparison between the concentration of parameters in the control and positive groups. * = concentration in the extract, normalized by weight; ** = concentration normalized to creatinine; SD = standard deviation; LDL = low density lipoprotein; TC = total cholesterol; HDL = high density lipoprotein; and TG = triglycerides.

Parameter		LDL	TC	HDL	TG	Prolactin	Citric Acid (ng/mL)		
		mg/dL				ng/mL	Serum	Tissue *	Urine **
Control Group		136.10 ± 42.72	207.60 ± 43.82	53.67 ± 11.22	119.00 ± 54.80	8.56 ± 3.40	1.10 ± 0.48	0.35 ± 0.14	0.012 ± 0.002
PCa Patients	pT2a 2b	122.80 ± 27.73	185.00 ± 40.70	50.00 ± 16.00	154.20 ± 66.03	18.20 ± 43.80	1.30 ± 0.32	0.20 ± 0.10	0.03 ± 0.03
	pT2c	131.80 ± 34.12	194.60 ± 44.99	51.22 ± 15.84	148.80 ± 69.42	13.73 ± 26.39	1.40 ± 0.39	0.21 ± 0.13	0.03 ± 0.03
	pT3	138.40 ± 34.44	201.90 ± 51.34	49.66 ± 19.81	165.90 ± 80.89	13.99 ± 18.86	1.40 ± 0.38	0.28 ± 0.23	0.02 ± 0.02
	Gleason < 7	132.20 ± 33.51	193.10 ± 50.84	51.00 ± 16.00	162.50 ± 81.69	19.34 ± 48.33	1.50 ± 0.38	0.18 ± 0.11	0.03 ± 0.03
	Gleason = 7	131.40 ± 34.07	199.30 ± 41.43	53.84 ± 17.59	150.40 ± 68.47	11.51 ± 13.52	1.30 ± 0.39	0.26 ± 0.19	0.03 ± 0.03
	Gleason > 7	131.60 ± 33.32	185.60 ± 50.34	42.95 ± 14.45	152.80 ± 70.47	15.93 ± 22.79	1.30 ± 0.34	0.21 ± 0.14	0.02 ± 0.02
*p*-value		0.442	0.198	0.120	0.367	0.321	0.082	2.51 × 10^−5^	1.73 × 10^−3^

**Table 3:** Characteristic of groups in reference to morphological evaluation according to the Gleason scale (GS) and pTNM stage assessment (B) characteristic of population in the two groups. PCa = prostate cancer cases and BPH = benign prostatic hyperplasia.

Samples	Urine	Tissue	Serum
PCa	134	132	109
Gleason Score (N%)			
GS < 7	33 (25)	33(25)	25 (23)
GS = 7	69 (51)	63 (48)	56 (51)
GS > 7	32 (24)	36 (27)	28 (26)
Pathological State (%)	135	131	109
Stage 2 A,B	23 (17)	24 (18)	22 (20)
Stage 2 C	77 (57)	74 (57)	61 (56)
Stage 3	35 (26)	33 (25)	26 (24)
Control	17	28	9

**Table 4:** Characteristic of groups in reference to morphological evaluation according to pTNM stage assessment characteristic of population in the two groups. PCa = prostate cancer cases and BPH = benign prostatic hyperplasia.

Parameter	Control	PCa
Number of Cases	30	160
Age (years)		
Mean ± SD	68.0 ± 7.1	69.0 ± 6.1
Range	54–79	48–80
Total PSA (ng ML^−1^)		
Mean ± SD	6.34 ± 3.82	14.80 ± 12.20
Range	0.868–18	0.008–45.53
Prostate Weight (g)		
Mean ± SD		43.6 ± 16.3
Range		21–139

## Data Availability

The data presented in this study are available on request from the corresponding author. The data are not publicly available due to law of Nicolaus Copernicus University in Toruń regarding the data storing.
